# Crystal structure of *N*-allyl-4-methyl­benzene­sulfonamide

**DOI:** 10.1107/S2056989018010290

**Published:** 2018-07-20

**Authors:** Zeel S. Patel, Amanda C. Stevens, Erin C. Bookout, Richard J. Staples, Shannon M. Biros, Felix N. Ngassa

**Affiliations:** a Department of Chemistry, Grand Valley State University, 1 Campus Dr., Allendale, MI 49401, USA; b Center for Crystallographic Research, Department of Chemistry, Michigan State University, East Lansing, MI 48824, USA

**Keywords:** crystal structure, aryl sulfonamide, hydrogen bond, offset π–π inter­action

## Abstract

The title compound, C_10_H_13_NO_2_S, was synthesized by a nucleophilic substitution reaction between allyl amine and *p*-toluene­sulfonyl chloride. The sulfonate S—O bond lengths are 1.4282 (17) and 1.4353 (17) Å, and the C—N—S—C torsion angle involving the sulfonamide moiety is −61.0 (2)°. In the crystal, centrosymmetric dimers of the title compound are present *via* inter­molecular N—H⋯O hydrogen bonds between sulfonamide groups. These dimers are linked into ribbons along the *c*-axis direction through offset π–π inter­actions.

## Abstract 2

In the crystal structure of the title sulfonamide, inter­molecular N—H⋯O hydrogen bonds are present between sulfonamide groups, as well as offset π–π inter­actions.

## Chemical context   

The sulfonamide moiety has been widely studied and its application in drug design has been reported (Qadir *et al.*, 2015 ▸; Rehman *et al.*, 2017 ▸; Gul *et al.*, 2018 ▸). Sulfa drugs, which incorporate the sulfonamide moiety, have found applications as anti­bacterial, anti­cancer, anti­fungal, anti-inflammatory, and anti­viral agents (Alaoui *et al.*, 2017 ▸).

The synthesis of sulfonamides generally relies on the use of sulfonyl chlorides as electrophilic partners that react with nucleophilic amines. According to the current state of knowledge in the field, the use of sulfonyl chlorides as electrophilic substrates in the synthesis of sulfonamides suffers from some drawbacks. One such drawback is the difficulty in handling and storage (Caddick *et al.*, 2004 ▸). Other alternatives to sulfonyl chlorides have been reported (Parumala & Peddinti, 2016 ▸; Yang & Tian, 2017 ▸). Nucleophilic acyl substitution is the mechanism that describes the reaction between a carb­oxy­lic acid derivative such as acid chloride with an amine to form the corresponding amide. The mechanism of the reaction between sulfonyl chlorides and amines is analogous to nucleophilic acyl substitution, except that it occurs at the sulfonyl group and not the carbonyl group (Um *et al.*, 2013 ▸).

**Figure 1:**
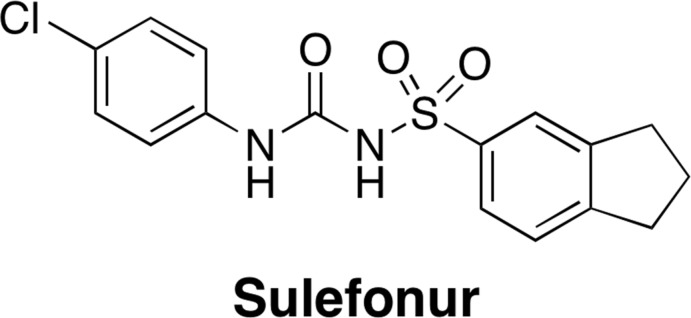
The structure of Sulefonur.

Recently, we have been particularly inter­ested in the structural motif of sulfonamide compounds that are known to modulate 5-HT_6_ receptor activity and are used for the treatment of CNS diseases and disorders (Blass, 2016 ▸). We are also inter­ested in the therapeutic application of sulfonamide mol­ecules used for chondrogenic differentiation (Choi *et al.*, 2016 ▸), and for the treatment of cancer (Gul *et al.*, 2018 ▸). Fig. 1 ▸ shows the structure of Sulefonur, which has been reported as a potent anti­cancer sulfonamide drug candidate and is under anti­cancer clinical trials (Gul *et al.*, 2018 ▸). As part of our ongoing effort to synthesize small sulfonamide mol­ecules that mimic the structural motifs of known sulfonamide drug candidates, we synthesized the title compound, C_10_H_13_NO_2_S, (I) and determined its crystal structure from single crystal X-ray diffraction data.
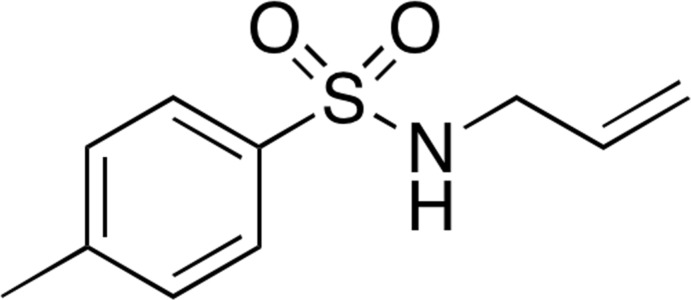


## Structural commentary   

**Figure 2:**
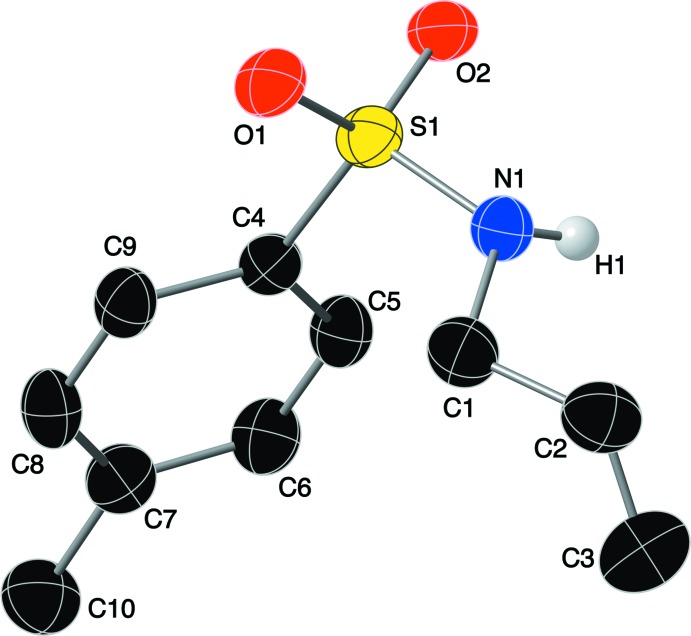
The mol­ecular structure of the title compound, showing the atom labeling. Displacement ellipsoids are drawn at the 50% probability level.

The mol­ecular structure of compound (I), which was solved in the triclinic space group *P* 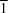, is shown in Fig. 2 ▸. The S—O bond lengths of 1.4282 (17) and 1.4353 (17) Å and the O1—S1—O2 bond angle of 118.87 (11)° are typical for sulfonamide moieties. The S1—N1 bond length is 1.617 (2) Å, and the C1—N1—S1—C4 torsion angle is −61.0 (2)°.

## Supra­molecular features   

**Figure 3:**
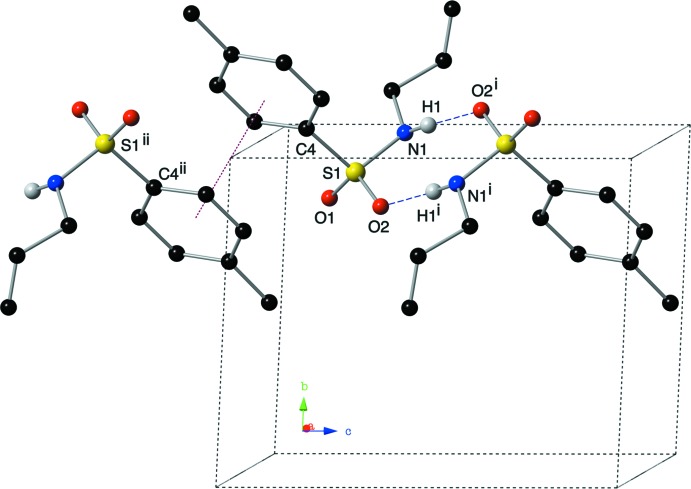
A depiction of the inter­molecular hydrogen bonds and offset π–π inter­actions present in the crystal, viewed down the *a* axis, using a ball and stick model with standard CPK colors. [Symmetry codes: (i) −*x*, −*y*, −*z* + 1; (ii) −*x*, −*y*, −*z*.]

Mol­ecules of the title compound are linked to one another *via* hydrogen bonds and π–π inter­actions. Centrosymmetric dimers of compound (I) are formed through inter­molecular hydrogen bonds between the sulfonamide N—H group and an O atom of a neighbouring sulfonamide group (Fig. 3 ▸). The N1⋯O2^i^ distance of 2.900 (3) Å suggests inter­actions of medium strength with a nearly linear N—H⋯O hydrogen bond of 174 (3)° (Table 1 ▸). These dimers are then linked through offset π–π inter­actions into ribbons that lie along the *c* axis (Figs. 3 ▸, 4 ▸). The inter­centroid distance *Cg*⋯*Cg* ^ii^ is 3.8340 (17) Å, with a slippage of 1.320 Å and a plane-to-plane distance between phenyl rings of 3.600 Å [symmetry code (ii) = −*x*, −*y*, −*z*].

## Database survey   

The Cambridge Structural Database (CSD, Version 5.39, February 2018; Groom *et al.*, 2016 ▸) contains 17 structures of *p*-tolyl­sulfonamides where there is a –CH_2_—C=C group bonded to the sulfonamide-N atom. The alkene group in these structures is a part of, for example, furan rings (DERTIE and DERTOK, Hashmi *et al.*, 2006 ▸), an allene (XUDNEP, Lan & Hammond, 2002 ▸), and various acyclic systems (BUXYUQ, Kiyokawa *et al.*, 2015 ▸; KIHMIY, Lee *et al.*, 2007 ▸). While all of the structures listed here display inter­molecular hydrogen bonds between sulfonamide groups, none of them display π–π inter­actions between the *p*-tolyl­sulfonamide rings as seen in the title compound.

## Synthesis and crystallization   

Allyl­amine (1.31 ml, 18 mmol) was added in 20 ml of degassed di­chloro­methane. This was followed by the addition of pyridine (1.42 ml, 18 mmol). The resulting solution was stirred under an atmosphere of N_2_, followed by the portion-wise addition of *p*-toluene­sulfonyl chloride (3.05 g, 16 mmol). The mixture was stirred at room temperature for 24 h. Reaction completion was verified by using TLC analysis. The mixture was acidified to pH 2–3 using concentrated HCl. After dilution with 20 ml of CH_2_Cl_2_, the organic phase was washed with H_2_O (3 × 20 ml) and the aqueous layer was back-extracted with CH_2_Cl_2_ (20 ml). The combined organic extracts were dried over anhydrous Na_2_SO_4_. After solvent evaporation, the residue was obtained as a yellow solid which was recrystallized in cold ethanol to afford pale-yellow crystals (56%; m.p. 332–333 K).

## Refinement   

Crystal data, data collection and structure refinement details are summarized in Table 2 ▸. All hydrogen atoms bonded to carbon atoms were placed in calculated positions and refined as riding: C*sp* ^3^—H = 0.95–1.00 Å with *U*_iso_(H) = 1.2*U*_eq_(C) for methine and methyl­ene groups, and *U*_iso_(H) = 1.5*U*_eq_(C) for methyl groups. The hydrogen atom bonded to the nitro­gen atom (H1) was located using electron-density difference maps, and the N—H bond length was restrained to 0.84±0.01 Å using the DFIX command as executed in *SHELXL* (Sheldrick, 2015 ▸).

## Supplementary Material

Crystal structure: contains datablock(s) I. DOI: 10.1107/S2056989018010290/wm5455sup1.cif

Structure factors: contains datablock(s) I. DOI: 10.1107/S2056989018010290/wm5455Isup2.hkl

Supporting information file. DOI: 10.1107/S2056989018010290/wm5455Isup3.cml

CCDC reference: 1856234

Additional supporting information:  crystallographic information; 3D view; checkCIF report

## Figures and Tables

**Figure 4:**
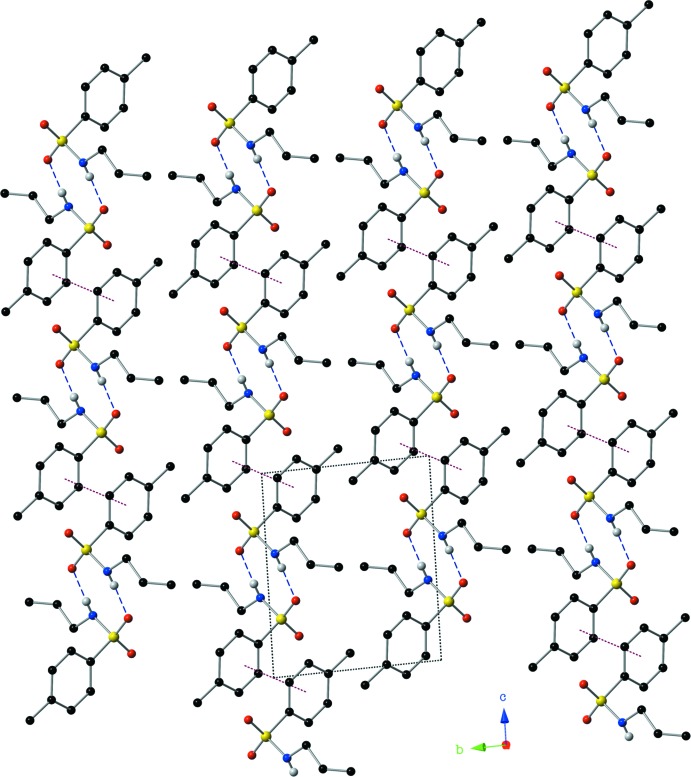
A view along the *a* axis of the title compound showing the supra­molecular ribbons assembled *via* N—H⋯O hydrogen bonds (blue, dashed lines) and π–π inter­actions (red, dotted lines).

**Table 1:** Hydrogen-bond geometry (Å, °)

*D*—H⋯*A*	*D*—H	H⋯*A*	*D*⋯*A*	*D*—H⋯*A*
N1—H1⋯O2^i^	0.83 (1)	2.07 (1)	2.900 (3)	174 (3)

Symmetry code: (i) 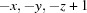.

**Table 2:** Experimental details

Crystal data	
Chemical formula	C_10_H_13_NO_2_S
*M*_r_	211.27
Crystal system, space group	Triclinic, *P* 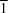
Temperature (K)	173
*a*, *b*, *c* (Å)	7.5538 (10), 8.2591 (11), 9.7145 (13)
α, β, γ (°)	85.9415 (16), 72.9167 (16), 67.6989 (15)
*V* (Å^3^)	535.42 (12)
*Z*	2
Radiation type	Mo *K*α
μ (mm^−1^)	0.28
Crystal size (mm)	0.28 × 0.25 × 0.20

Data collection	
Diffractometer	Bruker APEXII CCD
Absorption correction	Multi-scan (*SADABS*; Bruker, 2014 ▸)
*T*_min_, *T*_max_	0.672, 0.745
No. of measured, independent and observed [*I* > 2σ(*I*)] reflections	6472, 1963, 1564
*R*_int_	0.036
(sin θ/λ)_max_ (Å^−1^)	0.604

Refinement	
*R*[*F* ^2^ > 2σ(*F* ^2^)], *wR*(*F* ^2^), *S*	0.053, 0.156, 1.09
No. of reflections	1963
No. of parameters	132
No. of restraints	1
H-atom treatment	H atoms treated by a mixture of independent and constrained refinement
Δρ_max_, Δρ_min_ (e Å^−3^)	0.60, −0.26

Computer programs: *APEX2* and *SAINT* (Bruker, 2013 ▸), *SHELXS* (Sheldrick, 2008 ▸), *SHELXL* (Sheldrick, 2015 ▸), *OLEX2* (Dolomanov *et al.*, 2009 ▸; Bourhis *et al.*, 2015 ▸) and *CrystalMaker* (Palmer, 2007 ▸).

## supplementary crystallographic information

### Crystal data

**Table:**

C_10_H_13_NO_2_S	*Z* = 2
*M**_r_* = 211.27	*F*(000) = 224
Triclinic, *P*1	*D*_x_ = 1.310 Mg m^−^^3^
*a* = 7.5538 (10) Å	Mo *K*α radiation, λ = 0.71073 Å
*b* = 8.2591 (11) Å	Cell parameters from 2805 reflections
*c* = 9.7145 (13) Å	θ = 2.2–25.3°
α = 85.9415 (16)°	µ = 0.28 mm^−^^1^
β = 72.9167 (16)°	*T* = 173 K
γ = 67.6989 (15)°	Chunk, pale yellow
*V* = 535.42 (12) Å^3^	0.28 × 0.25 × 0.20 mm

### Data collection

**Table:**

Bruker APEXII CCD diffractometer	1564 reflections with *I* > 2σ(*I*)
φ and ω scans	*R*_int_ = 0.036
Absorption correction: multi-scan (*SADABS*; Bruker, 2014)	θ_max_ = 25.4°, θ_min_ = 2.2°
*T*_min_ = 0.672, *T*_max_ = 0.745	*h* = −9→9
6472 measured reflections	*k* = −9→9
1963 independent reflections	*l* = −11→11

### Refinement

**Table:**

Refinement on *F*^2^	Primary atom site location: structure-invariant direct methods
Least-squares matrix: full	Hydrogen site location: mixed
*R*[*F*^2^ > 2σ(*F*^2^)] = 0.053	H atoms treated by a mixture of independent and constrained refinement
*wR*(*F*^2^) = 0.156	*w* = 1/[σ^2^(*F*_o_^2^) + (0.0902*P*)^2^ + 0.112*P*] where *P* = (*F*_o_^2^ + 2*F*_c_^2^)/3
*S* = 1.09	(Δ/σ)_max_ < 0.001
1963 reflections	Δρ_max_ = 0.60 e Å^−^^3^
132 parameters	Δρ_min_ = −0.26 e Å^−^^3^
1 restraint	

### Special details

**Table:**

Geometry. All esds (except the esd in the dihedral angle between two l.s. planes) are estimated using the full covariance matrix. The cell esds are taken into account individually in the estimation of esds in distances, angles and torsion angles; correlations between esds in cell parameters are only used when they are defined by crystal symmetry. An approximate (isotropic) treatment of cell esds is used for estimating esds involving l.s. planes.

### Fractional atomic coordinates and isotropic or equivalent isotropic displacement parameters (Å^2^)

**Table:**

	*x*	*y*	*z*	*U*_iso_*/*U*_eq_	
S1	0.23035 (9)	−0.06592 (8)	0.28093 (6)	0.0355 (3)	
O1	0.4258 (2)	−0.1649 (2)	0.19086 (19)	0.0423 (5)	
O2	0.1100 (3)	−0.1576 (2)	0.36436 (18)	0.0407 (5)	
N1	0.2532 (3)	0.0508 (3)	0.3966 (2)	0.0352 (5)	
H1	0.153 (3)	0.085 (4)	0.468 (2)	0.048 (8)*	
C1	0.3608 (4)	0.1687 (4)	0.3441 (3)	0.0436 (7)	
H1A	0.2923	0.2565	0.2830	0.052*	
H1B	0.4984	0.1006	0.2847	0.052*	
C2	0.3677 (4)	0.2590 (4)	0.4684 (3)	0.0483 (7)	
H2	0.4323	0.1874	0.5339	0.058*	
C3	0.2948 (6)	0.4239 (5)	0.4948 (4)	0.0705 (10)	
H3A	0.2289	0.5005	0.4322	0.085*	
H3B	0.3063	0.4703	0.5770	0.085*	
C4	0.0966 (3)	0.0803 (3)	0.1729 (3)	0.0318 (6)	
C5	−0.1007 (4)	0.1904 (3)	0.2347 (3)	0.0390 (6)	
H5	−0.1655	0.1827	0.3334	0.047*	
C6	−0.2011 (4)	0.3105 (4)	0.1516 (3)	0.0424 (7)	
H6	−0.3363	0.3849	0.1937	0.051*	
C7	−0.1092 (4)	0.3257 (3)	0.0072 (3)	0.0397 (6)	
C8	0.0869 (4)	0.2125 (4)	−0.0528 (3)	0.0423 (7)	
H8	0.1509	0.2187	−0.1520	0.051*	
C9	0.1909 (4)	0.0909 (4)	0.0291 (3)	0.0380 (6)	
H9	0.3258	0.0156	−0.0130	0.046*	
C10	−0.2180 (5)	0.4608 (4)	−0.0817 (3)	0.0506 (7)	
H10A	−0.1212	0.4891	−0.1614	0.076*	
H10B	−0.3076	0.5669	−0.0212	0.076*	
H10C	−0.2964	0.4147	−0.1205	0.076*	

### Atomic displacement parameters (Å^2^)

**Table:**

	*U*^11^	*U*^22^	*U*^33^	*U*^12^	*U*^13^	*U*^23^
S1	0.0316 (4)	0.0323 (4)	0.0372 (4)	−0.0107 (3)	−0.0031 (3)	−0.0010 (3)
O1	0.0323 (10)	0.0378 (10)	0.0444 (10)	−0.0067 (8)	−0.0007 (8)	−0.0047 (8)
O2	0.0425 (10)	0.0322 (10)	0.0428 (10)	−0.0158 (9)	−0.0034 (8)	0.0010 (8)
N1	0.0325 (11)	0.0363 (12)	0.0333 (11)	−0.0124 (10)	−0.0049 (9)	0.0010 (9)
C1	0.0464 (16)	0.0444 (16)	0.0430 (15)	−0.0237 (13)	−0.0087 (12)	0.0036 (12)
C2	0.0484 (17)	0.0476 (18)	0.0550 (17)	−0.0218 (14)	−0.0196 (14)	0.0063 (14)
C3	0.084 (3)	0.056 (2)	0.073 (2)	−0.0282 (19)	−0.020 (2)	−0.0084 (18)
C4	0.0280 (12)	0.0331 (13)	0.0346 (13)	−0.0140 (11)	−0.0058 (10)	−0.0009 (10)
C5	0.0337 (14)	0.0439 (16)	0.0344 (13)	−0.0135 (12)	−0.0032 (11)	−0.0019 (12)
C6	0.0334 (14)	0.0474 (17)	0.0421 (15)	−0.0113 (12)	−0.0086 (12)	−0.0027 (13)
C7	0.0431 (15)	0.0423 (16)	0.0433 (15)	−0.0228 (13)	−0.0173 (12)	0.0013 (12)
C8	0.0415 (15)	0.0546 (17)	0.0312 (13)	−0.0227 (14)	−0.0045 (11)	0.0008 (12)
C9	0.0339 (14)	0.0442 (15)	0.0337 (13)	−0.0169 (12)	−0.0022 (11)	−0.0038 (11)
C10	0.0553 (18)	0.0505 (18)	0.0528 (17)	−0.0216 (15)	−0.0242 (14)	0.0076 (14)

### Geometric parameters (Å, º)

**Table:**

S1—O1	1.4282 (17)	C4—C9	1.383 (3)
S1—O2	1.4353 (17)	C5—H5	0.9500
S1—N1	1.617 (2)	C5—C6	1.373 (4)
S1—C4	1.760 (3)	C6—H6	0.9500
N1—H1	0.831 (10)	C6—C7	1.390 (4)
N1—C1	1.468 (3)	C7—C8	1.388 (3)
C1—H1A	0.9900	C7—C10	1.501 (4)
C1—H1B	0.9900	C8—H8	0.9500
C1—C2	1.487 (4)	C8—C9	1.383 (4)
C2—H2	0.9500	C9—H9	0.9500
C2—C3	1.273 (4)	C10—H10A	0.9800
C3—H3A	0.9500	C10—H10B	0.9800
C3—H3B	0.9500	C10—H10C	0.9800
C4—C5	1.390 (3)		
			
O1—S1—O2	118.87 (11)	C9—C4—C5	120.4 (2)
O1—S1—N1	107.94 (11)	C4—C5—H5	120.3
O1—S1—C4	108.08 (11)	C6—C5—C4	119.3 (2)
O2—S1—N1	105.56 (11)	C6—C5—H5	120.3
O2—S1—C4	108.64 (11)	C5—C6—H6	119.3
N1—S1—C4	107.21 (11)	C5—C6—C7	121.5 (2)
S1—N1—H1	112 (2)	C7—C6—H6	119.3
C1—N1—S1	119.02 (17)	C6—C7—C10	121.1 (3)
C1—N1—H1	118 (2)	C8—C7—C6	118.2 (2)
N1—C1—H1A	109.7	C8—C7—C10	120.7 (2)
N1—C1—H1B	109.7	C7—C8—H8	119.4
N1—C1—C2	109.8 (2)	C9—C8—C7	121.2 (2)
H1A—C1—H1B	108.2	C9—C8—H8	119.4
C2—C1—H1A	109.7	C4—C9—C8	119.3 (2)
C2—C1—H1B	109.7	C4—C9—H9	120.3
C1—C2—H2	117.1	C8—C9—H9	120.3
C3—C2—C1	125.7 (3)	C7—C10—H10A	109.5
C3—C2—H2	117.1	C7—C10—H10B	109.5
C2—C3—H3A	120.0	C7—C10—H10C	109.5
C2—C3—H3B	120.0	H10A—C10—H10B	109.5
H3A—C3—H3B	120.0	H10A—C10—H10C	109.5
C5—C4—S1	119.60 (19)	H10B—C10—H10C	109.5
C9—C4—S1	119.9 (2)		

### Hydrogen-bond geometry (Å, º)

**Table:**

*D*—H···*A*	*D*—H	H···*A*	*D*···*A*	*D*—H···*A*
N1—H1···O2^i^	0.83 (1)	2.07 (1)	2.900 (3)	174 (3)

Symmetry code: (i) −*x*, −*y*, −*z*+1.
